# Association between sociodemographic factors and mobility limitation among older adults: a systematic review and meta-analysis protocol

**DOI:** 10.1186/s13643-023-02190-9

**Published:** 2023-02-14

**Authors:** Ogochukwu Kelechi Onyeso, Adesola C. Odole, David R. Scott, Olayinka Akinrolie, Michael E. Kalu, Oluwagbohunmi A. Awosoga

**Affiliations:** 1 Faculty of Health Sciences, University of Lethbridge, Lethbridge, AB Canada; 2 Emerging Researchers and Professionals in Ageing-African Network, Abuja, Nigeria; 3 Prentice Institute for Global Population and Economy, University of Lethbridge, Lethbridge, AB Canada; 4 Department of Physiotherapy, Faculty of Clinical Sciences, College of Medicine, University of Ibadan, Ibadan, Oyo Nigeria; 5 University of Lethbridge Library, Lethbridge, AB Canada; 6 Applied Health Sciences, Faculty of Graduate Studies, University of Manitoba, Winnipeg, MB Canada; 7 School of Rehabilitation Science, McMaster University, Hamilton, ON Canada

## Abstract

**Background:**

Mobility is an independent predictor of physical functionality, healthy ageing, and quality of life. Various literatures have associated mobility limitation in older adulthood with demographic and socioeconomic factors. Hence, we propose a systematic review and meta-analysis to synthesise the association between sociodemographic factors and mobility limitations in older adults.

**Methods and analyses:**

This protocol was written according to the Preferred Reporting Items for Systematic Review and Meta-Analysis Protocols (PRISMA-P) guidelines. We will perform a comprehensive search of all observational studies that assessed the relationship between age, gender, race, place, education, income, occupation, social status, and walking distance, time, or speed. Electronic databases (MEDLINE, Web of Science, EMBASE, CINAHL, AgeLine, and SPORTDiscus) will be searched from inception to 28 February 2023. We will supplement the database search by manually searching the reference lists of all identified and relevant full-text articles. Two independent reviewers will be responsible for screening articles, data extraction, and assessment of bias. We will appraise the study quality and risk of bias using the Prediction Model Risk of Bias Assessment Tool (PROBAST). A meta-analysis will be considered if data from the selected studies are homogeneous, otherwise, a narrative synthesis of the extracted data will be presented.

**Discussion:**

Mobility limitation leads to frequent falls, dependency, morbidity, and death among older adults. This review is necessary, to identify and prioritise important sociodemographic factors during older adults’ clinical assessment and policy development. It is the first phase of a multi-methods study seeking to develop a prognostic mobility trajectory for community-dwelling older adults.

**Systematic review registration:**

PROSPERO CRD42022298570

**Supplementary Information:**

The online version contains supplementary material available at 10.1186/s13643-023-02190-9.

## Background

Mobility is a broad term with diverse contextual meanings [1, 2]. In this review protocol, we defined mobility as a person’s ability to move around safely and independently with or without a walking aid [3]. Mobility is fundamental to active ageing, health status, and quality of life [1, 2, 4]. Mobility limitation is an early predictor of physical disability [5], leading to frequent falls, dependency, and death among older adults [2–4]. The prevalence of mobility limitation among older adults ranged from 22.5 to 46.5%, in developed countries [6]. The World Health Organisation defined older adults as people aged 60 years and above [7]. Older adults and researchers are keenly interested in understanding the factors that influence mobility and ways to maximise movement potential in older adulthood [1, 2, 8].

Webber and colleagues have conceptualised a comprehensive older adults’ mobility model including cognitive, environmental, financial, personal, physical, psychological, and social factors [2]. However, biophysical, and psycho-cognitive aspects of older adults’ mobility are more frequently researched, creating a literature gap on the implications of the sociodemographic factors [9]. Exploring the influences of demographic and socioeconomic factors may further our understanding of the risks of mobility limitation in older adults. The demographic factors to be considered in this review are age, gender, race, and location [10, 11], while the socioeconomic determinants will be income, occupation, education, and social status [12–16].

Knowledge of the impact of sociodemographic factors on older adults’ mobility would assist clinicians and policymakers to develop strategies for the management of mobility decline among older adults. We propose to conduct the first systematic review of the association between sociodemographic factors and mobility in older adults. A recent scoping review [9] examined the effect of social interactions, cognition, and psychological factors on older adults’ mobility without emphasis on sociodemographic determinants. Therefore, we propose to conduct this review to synthesise the association between sociodemographic factors and performance-based walking outcomes including walking distance, time, and speed among community-dwelling older adults.

### Review questions

1. What is the direction of the association between sociodemographic factors and walking outcomes among community-dwelling older adults as reported in literature published from 1946 to 2023?
2. What is the size of the association between sociodemographic factors and walking outcomes among community-dwelling older adults as reported in literature published from 1946 to 2023?

### PECOT criteria

The *population* of the review will include community-dwelling older adults aged 60 years and older. The *exposure* will include sociodemographic factors: age, gender, race, location, income, occupation, education, and social status [16]. The *primary outcome* will include performance-based walking parameters such as walking distance, time, and speed. Outcome measures will include timed-up and go (TUG), short physical performance battery (SPPB), 6-minute walk test (6MWT), ten-metre walk test (10MWT), habitual gait speed (HGS), and backwards walking (BW) [1]. The review will cover the time between the inception of the oldest database and 2023 (1946 to 28 February 2023).

### Systematic review team members

The primary investigator (OKO) will organise and coordinate this review process: development of the research questions, search strategies, screening of relevant articles, data extraction and analyses, and manuscript preparation. The content expert will include review authors OAA, ACO, and MEK (who are experts in older adults’ mobility). Two review authors OKO and MEK will independently screen the citation for article and abstract inclusion and OKO and OA will perform the full-text screening and independently extract data from the included studies. A subject librarian (DRS) who is an expert in systematic review search methodology and OKO will develop the search strategies for all the included databases and conduct the literature search. Literature synthesis will be completed by OKO, OA, and MEK who are knowledgeable in systematic and scoping reviews. Statistical analysis will be done by OAA and ACO who are experts in meta-analysis.

## Methods

### Protocol and registration

This protocol has been registered within the International Prospective Register of Systematic Reviews (PROSPERO; registration number: CRD42022298570) [17]. This protocol was written according to the guidelines of the Preferred Reporting Items for Systematic Reviews and Meta-Analyses Protocols (PRISMA-P) [18]. We also adhered to the recommendations of Meta-analysis of Observational Studies in Epidemiology (MOOSE) [19]. The PRISMA-P checklist is given in Additional file 1. Any amendments to this protocol will be documented and published alongside the results of the systematic review.

### Eligibility criteria

#### Inclusion criteria

Studies will be included if they (1) were observational studies evaluating mobility in apparently healthy community-dwelling older adults (≥ 60 years), (2) described an association between any of the sociodemographic variables and walking parameters identified in this study, (3) were published between 1946 and 28 February 2023, and (4) were published in the English language only. (5) There will be no restriction regarding publication country, race, and gender. To be included in the meta-analysis part of the study, a study should provide the zero-order associations or partial correlation between sociodemographic factors and walking parameters or provide sufficient information for these associations (effect sizes) to be calculated and transformed into odds ratios [20].

#### Exclusion criteria

Studies will be excluded if focused on older adults (1) that were non-ambulatory, (2) with cardiopulmonary, cognitive, or neuromuscular diseases such as disabling stroke, parkinsonism, Alzheimer’s disease, dementia, or chronic obstructive pulmonary disease, (3) residing in an institutionalised or continuing care facility, and (4) if the data is overlapping or a duplicate of an already included study (we will choose the article with least risk of bias and most recent publication date).

### Outcome measures

The outcomes will be walking distance, time, and speed measured with performance-based tests (PBTs) such as TUG, SPPB, 6MWT, UG, HGS, 10MWT, and BW [1]. Walking distance is defined as the distance (metres) covered during a timed walking test (e.g. 6MWT); walking time is the time taken (seconds) to complete a specific distance (e.g. 10MWT); and walking speed is defined as walk distance divide by walk time (m/s, e.g. HGS test).

### Information sources

Following Bramer and colleagues’ recommendation on electronic search databases combination [20], we intend to search Ovid MEDLINE, Web of Science, Ovid EMBASE, EBSCO CINAHL, EBSCO AgeLine, and EBSCO SPORTDiscus from inception to 28 February 2023. A draft MEDLINE search strategy developed by the subject librarian and the primary investigator was provided in Additional file 2.

### Search strategy development

Search terms were identified through consultations between the primary investigator, content experts, and the librarian, and a review of the titles and abstracts of six seed articles gathered by the primary investigator [10–15]. Elements of search strings developed for previously published reviews also informed the search strategy development [21–23].

The draft MEDLINE search strategy will be peer-reviewed by another librarian who is not part of this review and comments will be addressed. Afterwards, the search strategy will be translated into different syntaxes recognised by each database. Subject headings (e.g. MeSH), Boolean operators, proximity operators, truncation, and phrase searching will be used appropriately as shown in Additional file 2.

### Data management

The results of the search from the different databases will be exported to EndNote 20 (a citation manager) and duplicates will be removed. After removing duplicates, the articles will be exported to Rayyan—a web-based systematic review management tool [24] that will be used for the title, abstract, and full-text screening. Included and excluded articles will be exported to and organised in EndNote 20 for the generation of the PRISMA flow chart and in-text citations.

### Study selection and data extraction

We will adopt a two-stage screening (title and abstract screening, and full-text screening) to select eligible studies. At the two stages of screening, two review authors will independently screen for studies that are relevant to the objectives of this review using the selection criteria. There will be a pilot screening before the full-text screening. Two review authors (OKO and OA) will independently screen 50 studies; their results will be compared and resolved to maximise inter-reviewer agreement ahead of the full-text screening process. Similarly, data extraction will be done independently by two review authors (OKO and OA) and piloted on a small sample of selected studies using a standardised data extraction form set up on a Microsoft Excel spreadsheet (Additional file 3). Conflict arising through this process will be resolved by a third review author (OAA). Following the description by Lipsey and Wilson [25], and Khaliq and colleagues [26], we will extract the following information from each study: citation details such as first author, year and country of publication, study design (cohort, case-control, cross-sectional, or longitudinal study), sample size, participants’ demographic (age, sex/gender, race, location), socioeconomic factors studied (income, occupation, education, and social status), all the names of PBTs and other mobility assessment instruments used, the measured outcomes, and the statistical methods implemented including the descriptive summary of the outcomes and inferential results such correlation coefficients, odd ratios, relative risks, their effect sizes, and *p* values (see Additional file 3).

### Risk of bias and quality assessment

The Prediction Model Risk of Bias Assessment Tool (PROBAST) [27] will be used to assess the internal validity of the included studies. The study participation, attrition, risk factor measurement, outcome measurement, confounding factor, statistical analysis, and report completeness can be assessed and rated as low, moderate, or high risk of bias. Two review authors (OKO and OA) will complete the assessment independently and discrepancies will be resolved by another review author (OAA). The quality of evidence for the main outcome across the studies will be assessed using the GRADE approach [28], and rated high, moderate, low, or very low based on the confidence in the effect estimate, a summary of the risk of bias assessment, imprecision, and indirectness.

### Data analysis

#### Narrative synthesis

A narrative synthesis will be used to analyse the results of all included studies, and the association between socioeconomic factors and walking parameters will be classified by direction and strength: correlation coefficients < 0.3 will be interpreted as a weak association, 0.4 to 0.6 as a moderate association, and > 0.6 as a strong association [29]. Studies will be categorised according to the PBTs that were assessed [1], and the association between each primary outcome and any of the sociodemographic factors under review will be compared between studies that used the same PBT [30].

### Meta-analysis

Studies will be grouped according to their designs, such that, case-control, cohort, cross-sectional, and longitudinal studies will be analysed and reported separately [31]. Furthermore, due to anticipated sociodemographic changes over time, both aggregate (1946 to 2023) and separate analyses (20 years intervals; 1946 to 1966, 1967 to 1987, 1988 to 2008, 2009 to 2023) will be conducted for studies based on time-lagged data to determine the direction of associations over time. Comprehensive Meta-Analysis (CMA, version 3) software will be used to conduct the meta-analysis [32]. The overall synthesised measure of effect size will be reported with odd ratios (95% CI). The mean of the combined effect sizes will be calculated in studies where several effect sizes were reported from the same sample (e.g. models with different control variables) [33]. An overall estimate will be calculated for studies with overlapping samples. In studies reporting effect sizes from independent subgroups (e.g. moderators), each subgroup will be included as a unique sample in the meta-analysis. Moderation analyses will also be used to compare associations from cross-sectional and prospective data. The CMA weights studies by inverse variance [32], which is a method of aggregating multiple random variables where each random variable is weighted in inverse proportion to its variance to minimise the variance of the weighted average [33]. The inverse variance is approximately proportional to sample size, but it is a more nuanced measure and serves to minimise the variance of the combined effect [34].

As the individual studies included cannot be expected to come from the same population of studies, the pooled mean effect size will be calculated using the random-effects model [33]. Such effects models are thus recommended when accumulating data from a series of studies where the effect size is assumed to vary from one study to the next and where it is unlikely that studies are functionally equivalent [34]. Random effects models allow statistical inferences to be made to a population of studies beyond those included in the meta-analysis [35].

An *I*^2^ statistic will be computed as an indicator of heterogeneity in terms of percentages. Increasing values show increasing heterogeneity, with values of 0% indicating no heterogeneity, 50% indicating moderate heterogeneity, and 75% indicating high heterogeneity [36]. Therefore, we will not conduct a meta-analysis for any group of articles with *I*^2^ statistics greater than 75% [37]. However, we will attempt to fix the heterogeneity in groups with *I*^2^ statistics between 50% and 75% by checking and removing any outlier study. The “one-study-removed” procedure will be used as a sensitivity analysis to determine whether the overall estimates between sociodemographic factors and mobility limitations are influenced by outlier studies. Using this approach, effect sizes that fell outside the 95th confidence interval of the average effect size will be considered outliers [38]. Other ways we plan to reduce heterogeneity are by subgroup analysis based on study design and time lag, and the use of a random effect model as stated earlier [37]. Three indicators of publication bias are to be examined: funnel plot, Rosenthal’s Fail-Safe N, and Egger’s regression intercept. A Forest plot will be constructed for included studies.

## Discussion

The proposed systematic review will be impactful in the field of ageing research, clinical practice, policy formulation, and for the entire society. Gerontology has become a spotlight area due to the increase in population ageing, life expectancy, and age-related chronic diseases. By the next three decades, the global population of older adults will rise from eight hundred million to two billion people [39]. Thus, it can be predicted that age-related mobility limitations will increase the burden on social, economic, and healthcare systems [2, 4]. Most importantly, studies on older adults’ mobility have been biased towards the biophysical factors, yet sociodemographic determinant is a significant factor in access to health and healthy ageing [9]. This study will quantify and synthesise the various sociodemographic determinants of mobility in older adults as available in the literature so far and proffer recommendations on the critical directions for future research.

Mobility limitation is an early predictor of physical disability, leading to frequent falls, dependency, morbidity, and death among older adults [2, 4]. Geriatricians need this type of review to know the magnitude and direction of associations between sociodemographic factors and mobility decline. The outcome of this review will be necessary to develop prognostic models for geriatric care. Knowing the modifiable and non-modifiable determinants of mobility decline as envisaged in this study is fundamental for policy design and implementation [4].

The implication for society and policymakers is that all of us are ageing by the day, and the health of our aged dependents is our collective responsibility as a society. Social justice, equity, and fairness demand that our policies should be formed and implemented in cognisance of sociodemographic inequalities. National resources, infrastructures, basic amenities, and social welfare should be distributed in consideration of the real, perceive, and potential impacts of the mobility decline in the ageing population.

## Limitations

The meta-analysis will include observational studies, and the aggregated effect sizes will therefore not account for the cause-and-effect relationship between the included variables [33]. We may not be able to recruit at least two authors each that can perform screening and data extraction from articles written in languages other than English. This may lead to exclusion of some quality papers that met other inclusion criteria.

## Supplementary Information

**Additional file 1.** PRISMA-P (Preferred Reporting Items for Systematic Review and Meta-Analysis Protocols) 2015 checklist.

**Additional file 2.** MEDLINE search strategy for the systematic review.

**Additional file 3.** Data extraction sheet.

## Data Availability

Not applicable.

## Abbreviations

10MWT: Ten-metre walk test
6MWT: Six-minute walk test
BW: Backward walking
CINAHL: Cumulative Index to Nursing and Allied Health Literature
CMA: Comprehensive meta-analysis
EMBASE: Excerpta Medica Database
GRADE: Grading of Recommendations Assessment Development and Evaluation
HGS: Habitual gait speed
MEDLINE: Medical Literature Analysis and Retrieval System Online
MeSH: Medical Subject Headings
MOOSE: Meta-analysis of Observational Studies in Epidemiology
PBT: Performance-based tests
PRISMA: Preferred Reporting Items for Systematic Review and Meta-Analysis
PROBAST: Prediction model Risk of Bias Assessment Tool
PROSPERO: International Prospective Register of Systematic Reviews
SPPB: Short Physical Performance Battery
TUG: Timed Up and Go
UG: 8-Foot Up-and-Go
WHO: World Health Organization
