# Supplementary material for: Association between sociodemographic factors and mobility limitation among older adults: a systematic review and meta-analysis protocol
Source: Syst Rev. 2023 Feb 14;12:19. doi: 10.1186/s13643-023-02190-9 (PMC9930347; doi:10.1186/s13643-023-02190-9)
Supplement: Supplementary file 2 — Additional file 2. MEDLINE search strategy for the systematic review. [file 13643_2023_2190_MOESM2_ESM.docx]

**Additional file 2** MEDLINE search strategy for the systematic review

**MEDLINE Search Strategy for the Systematic Review**

1. exp Aged/ [MeSH]
2. (elderly or senior or seniors or "older adult" or "older adults" or geriatric or geriatrics or "old people" or older-age or "old age" or "older people").ti,ab.
3. or/1-2
4. Mobility Limitation/ [MeSH]
5. Walking Speed/ [MeSH]
6. ((walk* or gait* or ambulat* or locomot*) adj5 (speed* or pace* or difficult*)).ti,ab.
7. (mobilit* adj5 limit*).ti,ab.
8. or/4-7
9. Social Determinants of Health/ [MeSH]
10. exp Socioeconomic Factors/ [MeSH]
11. ((social or socioeconomic or economic or population*) adj3 (determinant* or factor* or risk* or equity or equities or inequit* or inequal* or equality or equalities or disparit*)).ti,ab.
12. (health adj3 (determinant* or equity or equities or inequit* or inequal* or equality or equalities or disparit*)).ti,ab.
13. "determinants of health".ti,ab.
14. exp Ethnic Groups/ [MeSH]
15. exp Continental Population Groups/ [MeSH]
16. (ethnic* or race or racial* or immigrant*).ti,ab.
17. exp Gender Identity/ [MeSH]
18. Sex/ [MeSH]
19. (gender* or sex).ti,ab.
20. exp Income/ [MeSH]
21. (((social or socioeconomic or economic) adj3 (status* or class*)) or income or poverty).ti,ab.
22. exp Employment/ [MeSH]
23. (employ* or unemploy* or occupation*).ti,ab.
24. or/9-23
25. 3 and 8 and 24
26. limit 25 to English language

Note: MeSH = medical subject heading; exp = used with a MeSH term to include all narrower MeSH terms; .ab, .ti = field codes for abstract and title, respectively; adj# = search for records with terms within # words of each other; * after keyword indicates truncation (e.g., ethnic* will retrieve “ethnic”, “ethnicity”, “ethnicities”, etc.)
