# Supplementary material for: Association between sociodemographic factors and mobility limitation among older adults: a systematic review and meta-analysis protocol
Source: Syst Rev. 2023 Feb 14;12:19. doi: 10.1186/s13643-023-02190-9 (PMC9930347; doi:10.1186/s13643-023-02190-9)
Supplement: Supplementary file 3 — Additional file 3. Data extraction sheet. [file 13643_2023_2190_MOESM3_ESM.docx]

**Additional file 3** Data extraction sheet

| 1 | 2 | 3 | 4 | 5 | 6 | 7 | 8 | 9 | 10 | 11 | 12 | 13 |
| --- | --- | --- | --- | --- | --- | --- | --- | --- | --- | --- | --- | --- |
| S/N | Authors/  Year/  Country | Study design  (dropdown) | Sample size | Age range | Descriptive Statistics/Sociodemographic characteristics | | | | | | | |
|  |  |  |  |  | Mean ± SD | | *f*(%) | | | | | |
|  |  |  |  |  | Age | Income | Gender | Race | Location | Occupation | Education | S. status |
|  |  | cohort, case-control, cross-sectional, or longitudinal |  | 60-64  65-69  70-74  75-79  ≥80  N/G | Years | Annual personal  income,  N/G | Females,  Males,  N/G | *Race, N/G | Rural,  Urban,  N/G | Skilled nonmanual,  Unskilled nonmanual,  Skilled manual,  Unskilled manual,  N/G | Informal,  Primary.  Secondary,  Tertiary,  N/G | High,  Middle,  Low,  N/G |
|  |  |  |  |  |  |  |  |  |  |  |  |  |

| 14 | 15 | 16 | 17 | 18 | 19 | 20 | 21 | 22 | 23 | 24 | 25 | 26 | 27 |
| --- | --- | --- | --- | --- | --- | --- | --- | --- | --- | --- | --- | --- | --- |
| PBT for primary outcome | Primary outcome | Secondary outcome/ instrument | Inferential Statistics | Correlation/association/OR/RR of primary outcome based on: | | | | | | | | p-value | Effect size |
|  |  |  |  | Age | Gender | Income | Race | Location | Occupation | Education | Social status |  |  |
| TUG, BW, 10MWT, SPPB, 6MWT, HGS | Walking speed, or  distance, or  Test time |  |  |  |  |  |  |  |  |  |  |  |  |
|  |  |  |  |  |  |  |  |  |  |  |  |  |  |

N/G = not given. PBT = performance-based test. S. status = social status. TUG = Timed Up and Go. SPPB = Short Physical Performance Battery. 6MWT = Six-Minute Walk Test. 10MWT = Ten-Minutes Walk Test. HGS = Habitual Gait Speed. BW = Backward Walking. *Race = American Indian, Asian, Black, Native Hawaiian or Other Pacific Islander, and White.
